# Development and application of a diagnostic and severity scale to grade post-operative pediatric cerebellar mutism syndrome

**DOI:** 10.1007/s00431-021-04290-x

**Published:** 2021-10-14

**Authors:** Federica S. Ricci, Rossella D’Alessandro, Alessandra Somà, Anna Salvalaggio, Francesca Rossi, Sara Rampone, Giorgia Gamberini, Chiara Davico, Paola Peretta, Mario Cacciacarne, Pierpaolo Gaglini, Paolo Pacca, Giulia Pilloni, Paola Ragazzi, Daniele Bertin, Stefano G. Vallero, Franca Fagioli, Benedetto Vitiello

**Affiliations:** 1 Section of Child and Adolescent Neuropsychiatry, Department of Public Health and Pediatric Sciences, University of Turin, Turin, Italy; 2 Child and Adolescent Neuropsychiatry Unit, Arrigo Hospital, Alessandria, Italy; 3 Section of Pediatric Neurosurgery, Children’s Hospital “Regina Margherita”, Torino, Italy; 4 Section of Pediatric Onco-Hematology, Department of Public Health and Pediatric Sciences, University of Turin, Turin, Italy; 5 Section of Child and Adolescent Neuropsychiatry, Children’s Hospital “Regina Margherita”, Piazza Polonia 94, 10126 Torino, Italy

**Keywords:** Posterior cranial fossa tumors, Pediatric cerebellar mutism syndrome, Pediatric cerebellar mutism syndrome scoring system

## Abstract

The post-operative pediatric cerebellar mutism syndrome (CMS) affects about one-third of children and adolescents following surgical removal of a posterior fossa tumor (PFT). According to the Posterior Fossa Society consensus working definition, CMS is characterized by delayed-onset mutism/reduced speech and emotional lability after cerebellar or 4th ventricle tumor surgery in children, and is frequently accompanied by additional features such as hypotonia and oropharyngeal dysfunction/dysphagia. The main objective of this work was to develop a diagnostic scale to grade CMS duration and severity. Thirty consecutively referred subjects, aged 1–17 years (median 8 years, IQR 3–10), were evaluated with the proposed Post-Operative Pediatric CMS Survey after surgical resection of a PFT and, in case of CMS, for 30 days after the onset (*T*_0_) or until symptom remission. At day 30 (*T*_1_), CMS was classified into mild, moderate, or severe according to the proposed scale. CMS occurred in 13 patients (43%, 95% C.I.: 25.5–62.6%), with mild severity in 4 cases (31%), moderate in 4 (31%), and severe in 5 (38%). At *T*_1_, longer symptom persistence was associated with greater severity (*p* = 0.01). Greater severity at *T*_0_ predicted greater severity at *T*_1_ (*p* = 0.0001). Children with a midline tumor location and those aged under 5 years at diagnosis were at higher risk of CMS (*p* = 0.025 and *p* = 0.008, respectively). In conclusion, the proposed scale is a simple and applicable tool for estimating the severity of CMS at its onset, monitoring its course over time, and providing an early prognostic stratification to guide treatment decisions.

**Supplementary Information:**

The online version contains supplementary material available at 10.1007/s00431-021-04290-x.

**Table:** 

**What is Known:** *• Post-operative pediatric Cerebellar Mutism Syndrome (CMS) is a complex phenomenon with a wide* *spectrum of symptoms that may manifest in children undergoing the resection of a posterior fossa tumor* *(PFT) and that can result into long-term impairment.*	
**What is New:** *• This study developed and pilot-tested an easily applicable diagnostic and severity scale to grade the* *duration and the severity of symptoms of the CMS.* *• The proposed scale was found to be a sensitive instrument to identify even mild CMS presentations.* *• By scoring not only the duration but also the severity of symptoms the scale allows a more accurate* *prognostic stratification for an optimal planning of clinical and rehabilitative interventions.*	

## Introduction

Post-operative pediatric cerebellar mutism syndrome (CMS) is a complex phenomenon with a wide spectrum of symptoms that may manifest with different combinations and severity in children undergoing surgical resection of a posterior fossa tumor (PFT). These children may present with impairments in linguistic, cognitive, motor, and affective/behavioral functioning. The core symptom of CMS is mutism or a severely reduced speech production, limited to single words or short sentences elicited only after vigorous stimulation [1]. On average, mutism occurs within 2 days post-operatively, but may not present itself for up to 7 days [2]. Although mutism is always transient and recovers spontaneously, long-term language impairments often persist [3]. Another remarkable feature of this condition is emotional lability, which is characterized by exaggerated changes in mood or affect with rapid fluctuation of emotional expression [4, 5].

The estimated incidence of CMS in children with PFT is between 11 and 30% [6]. Several risk factors have been identified: medulloblastoma tumor type, tumor location in the cerebellar midline or in the fourth ventricle, and brainstem involvement [7]. There are unconfirmed reports of other possible risk factors, including younger age, left-handedness [8], pre-operative language impairment [9, 10], and a lower socioeconomic background [11]. Other factors such as sex, pre-operative hydrocephalus, and extent of the resection do not seem to contribute to the risk of CMS [12]. For several years, confusion has encompassed the description of post-operative cerebellar mutism, as shown by the heterogeneous terminology used to describe the syndrome [6]. Moreover, no international guideline to date defines the diagnosis, prevention, treatment, or follow-up of this disabling condition [13]. For these reasons, in 2014, the Posterior Fossa Society (PFS), an international and multi-professional group of experts, was constituted to systematically gather and exchange information on the syndrome. In 2015, the PFS initiated an international consensus process to create a new shared definition of the condition, define standardized methods for the diagnosis and follow-up, and monitor late sequelae, as necessary steps towards ultimately improving the quality of life of pediatric brain tumor patients.

In the consensus paper, the PFS proposed a new working definition of the CMS: delayed onset mutism/reduced speech and emotional lability after cerebellar or 4th ventricle tumor surgery in children, with additional common features that include hypotonia and oropharyngeal dysfunction/dysphagia. It has been highlighted that the CMS may frequently be accompanied by the cerebellar motor syndrome, the cerebellar cognitive-affective syndrome, and brainstem dysfunction [13]. In this paper, the PFS pointed out the need for a new CMS scoring scale for the diagnosis of the syndrome, to measure not only the duration of symptoms, as in the CMS survey by [14], but also their severity, which is correlated with long-term impairment [14]. During the third Consensus Meeting of the PFS (Reykjavik, 2018), the need of a new evaluation scale of CMS was reiterated [15], possibly designed to be administrable as soon as possible after surgery, formulated in a simple and easy to complete way, and administrable by a variety of different health professionals. The importance of recording latency of onset, duration, and symptom severity was emphasized as a means of making a prognostic stratification and guiding treatment.

The aim of this work was to propose a diagnostic scale that grades the duration and severity of the symptoms of the CMS, and that is easily administered in clinical practice by different health professionals. Secondary aims were to estimate the incidence rate of CMS applying broader diagnostic criteria, and to define individual characteristics of patients with CMS in our sample.

## Materials and methods

### Study design

This was a single-center prospective cohort study on children who underwent surgery for a PFT. The study was carried out in accordance with the ethical principles enshrined in the Helsinki Declaration and was approved by the institution’s research ethics committee (Protocol N. 0,091,286). A written informed consent to study participation was signed by parents or legal guardians.

### Participants

Patients aged 1–17 years, admitted to the Pediatric Neurosurgery Unit of the Children’s Hospital “Regina Margherita” of Turin, Italy, between September 2017 and March 2021, with a diagnosis of a PFT, who underwent a neurosurgical intervention for total or partial tumor removal, or biopsy, were included. Patients with known pre-morbid neuropsychiatric diagnoses or severe complications in the post-operative period were excluded. The surgical reports were reviewed to determine the extent of the resection.

According to the standard protocol of the hospital, each patient received a comprehensive neurosurgical and neurological examination at the admission, and brain MRI scans during the pre-operative period. The neurosurgical removal operation was assisted by the intraoperative neurophysiological monitoring. In the post-operative period, each child received neurosurgical, neurological, and oncological examinations and MRI scans. In addition, the “Post-operative pediatric CMS survey” was completed immediately after surgery, and for the next 30 days or until symptoms remission. Symptom scoring was done after the observation of the patient by the attending physician or, if not possible, by other staff (i.e., nurses, physiotherapists). Family members assisting the child were asked to report and describe possible symptoms.

Patients’ demographics and neurological and language development history were collected. Pre-resection neurological functioning and speech were clinically assessed, and pre-resection MRI data (tumor localization, invasion or compression of the brainstem, and presence of hydrocephalus) were obtained. Post-resection clinical and MRI data, including the extent of the resection, were collected, together with histological results (according to the 2016 WHO System). The assessment of the extent of the tumor resection was based primarily on the post-operative MRI report and/or images, supplemented with the surgeon’s estimate, as detailed in the operative report. The extent of tumor resection was classified as follows: “Total”: the MRI and surgeon indicated complete resection; “Subtotal”: > 90% of the tumor removed; “Partial”: < 90% of the tumor removed; or “Biopsy”: no significant change in the size of tumor postoperatively. Hydrocephalus was defined as the presence of dilation of the ventricles on MRI and clinical signs and symptoms of increased intracranial pressure. The demographics and clinical characteristics of the sample are summarized in Tables 1 and 2.

**Table 1:** Patient characteristics and occurrence of CMS

**Variables**	**All patients **(***n = 30***)	**CMS **(***n = 13***)	**NO CMS **(***n = 17***)	P*
**Age at *T***_**0**_**, median (Q1–Q3)**	**8 **[3–10]	**4 **[2–8]	**9 **[7–13]	**0.004**
**Age at *T***_**0**_** ≤ 5 years old, *n* (%)**	**12 (40)**	**9 (69)**	**3 (18)**	**0.008**
Male sex, *n* (%)	19 (63)	7 (54)	12 (71)	0.45
Medulloblastoma, *n* (%)	6 (20)	3 (23)	3 (18)	1
Right hemisphere tumor location, *n* (%)	6 (20)	2 (15)	4 (23)	0.67
**Midline tumor location, *n* (%)**	**15 (50)**	**10 (77)**	**5 (29)**	**0.025**
Infiltration of brainstem, *n* (%)	8 (26)	3 (23)	5 (29)	1
Compression of brainstem, *n* (%)	12 (40)	5 (38)	7 (41)	1
Preoperative hydrocephalus, *n* (%)	25 (83)	11 (85)	14 (82)	0.62
Preoperative speech disorder, *n* (%)	7 (23)	3 (23)	4 (23)	1
Total tumor resection *n* = 29, (%)	18 (62)	9 (69)*	9 (53)	0.54

*T*_0_ time of CMS onset, *T*_1_ assessment at day 30th after CMS onset; *n tot = 12, 1 n.a

^*^Mann–Whitney *U* test, Fisher’s exact test or Fisher-Freeman-Halton test, as appropriate; *α* = 0.05

**Table 2:** Patients characteristics

**Variables**	**All patients (*n* = 30)**
Age at *T*_0_, median (Q1–Q3)	8 [3–10]
Age at *T*_0_ ≤ 5 years old, *n* (%)	**12 (40)**
Sex, *n* (%)	
Male	19 (63)
Female	11 (37)
Tumor histology *n* (%)	
Medulloblastoma	6 (20)
Pilocytic astrocytoma	15 (50)
Ependymoma	3 (10)
Diffused glioma	3 (10)
Cavernoma	1 (3)
Papilloma	1 (3)
Ganglioglioma	1 (3)
Tumor involvement, *n* (%)	
Left cerebellar hemisphere	9 (30)
Right cerebellar hemisphere	6 (20)
Midline (vermis/IV ventricle)	15 (50)
Brainstem involvement, *n* (%)	
Infiltration of brainstem	8 (26)
Compression of brainstem	12 (40)
Preoperative hydrocephalus, *n* (%)	25 (83)
Preoperative speech disorder, *n* (%)	7 (23)
Tumor resection *n* = 29* (%)	
Total	18 (62)
Partial	10 (35)
Biopsy	1 (3)

*T*_0_ time of CMS onset

^*^Data from 1 patient not available

### The CMS survey and scale

**Fig. 1:**
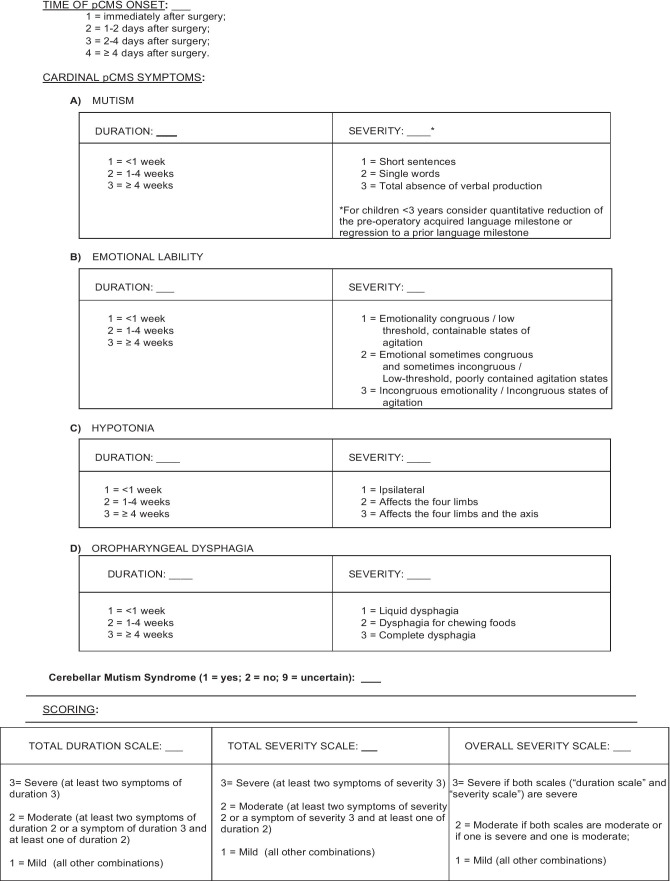
Pediatric Post-Operative Cerebellar Mutism Syndrome (CMS) Scale

The diagnostic and scoring scale for CMS was designed after a thorough review of the existing literature. The survey assesses the presence and the time of onset of the syndrome, as well as the duration and severity of the four symptoms, considering the latest definition of CMS as formulated by the PFS. Mutism and emotional lability (cardinal symptoms of the syndrome), hypotonia, and dysphagia (additional symptoms) were included in the survey. Mutism was considered as the only necessary and sufficient symptom for a CMS diagnosis. Not only the absence of speech but also a quantitative and qualitative change from pre-surgical speech production was classified as a manifestation of mutism ^13^. As suggested during the Consensus Meeting of 2018, the scoring system was first reviewed with the entire staff, to make sure that it was simple, clear, and feasible (Online Resource 1). The CMS survey was administered immediately after surgery and, if CMS occurred, for the next 30 days or until the complete remission of symptoms. For each of the four symptoms of the CMS Survey, a final score was assigned to its duration and severity. The severity final score corresponded to the highest score achieved in the first 30 days. A scoring system was proposed for assessing CMS duration and symptom intensity, both contributing to an overall CMS severity score (Fig. 1).

### Data analysis

Descriptive analyses were applied: categorical variables were expressed as absolute and percentage values, and continuous variables, as medians, with associated 1st quartile and 3rd quartile range (IQR). Based on the literature data [16, 17], age was dichotomized into two classes (class 1: less than/equal to 5 years and class 2: over 5 years). The variable “tumor type,” composed by seven classes of tumor histological types, was dichotomized for analytical purposes into “medulloblastoma/other etiology.” Similarly, the variable “tumor localization,” composed by five classes, was dichotomized into “involvement/non-involvement of midline structures (cerebellar worm, IV ventricle)”. Stratified data analyses were performed for CMS. The Fisher’s exact test or the Fisher-Freeman-Halton test, as appropriate, was used to measure associations between categorical variables. Mann–Whitney *U* test was used for the association analysis of the continuous variable “age at diagnosis.” Statistical significance was set at *α* ≤ 0.05 (two tailed). Statistical processing was performed using IBM SPSS Statistic software, version 25.0 (IBM Corp., Armonk, NY, USA).

## Results

### Sample demographics, clinical characteristics

Thirty-one children were enrolled in this study between September 2017 and March 2021. One patient was excluded because of severe complications in the post-operative period. Therefore, the sample consisted of 30 patients (19 males and 11 females), with a median age of 8 years (IQR 3–10) at *T*_0_. Twelve patients (40%) were aged under 5 years at the time of enrollment. Assessment of pre-operative speech behavior showed deficits in 7 patients (23%). The most common tumor types were pilocytic astrocytoma (50%), medulloblastoma (20%), diffuse astrocytoma (10%), and ependymoma (10%). The tumor was located at the midline in 15 cases (50%), with brainstem invasion in 8 children (27%) and a compression of it in 12 cases (40%). At the time of the PFT diagnosis, 25 patients (83%) had hydrocephalus (Table 2).

### CMS subgroup

CMS was diagnosed in 13 (43%, C.I. 95%: 25.5–62.6%) out of the 30 assessed patients (7 males and 6 females), with a median age of 4 years (IQR 2–8) at the onset of the symptoms (*T*_0_). Nine patients (69%) were under 5 years of age at diagnosis. Assessment of speech showed deficits in 3 (23%) patients. The CMS occurred in 3 out of 6 children with a diagnosis of medulloblastoma, 3 out of 3 with an ependymoma, 6 out of 15 with a pilocytic astrocytoma, and 1 out of 3 with diffuse astrocytoma. In 10 patients (77%), the tumor was located at the midline; in 3 children, the tumor invaded the brainstem (23%), and in 5 compressed it (38%). Eleven children (85%) had a preoperative hydrocephalus. Nine patients (69%) of the 13 children diagnosed with CMS had a complete resection and 4 had residual tumor.

### Comparison between patients with CMS and without CMS

**Fig. 2:**
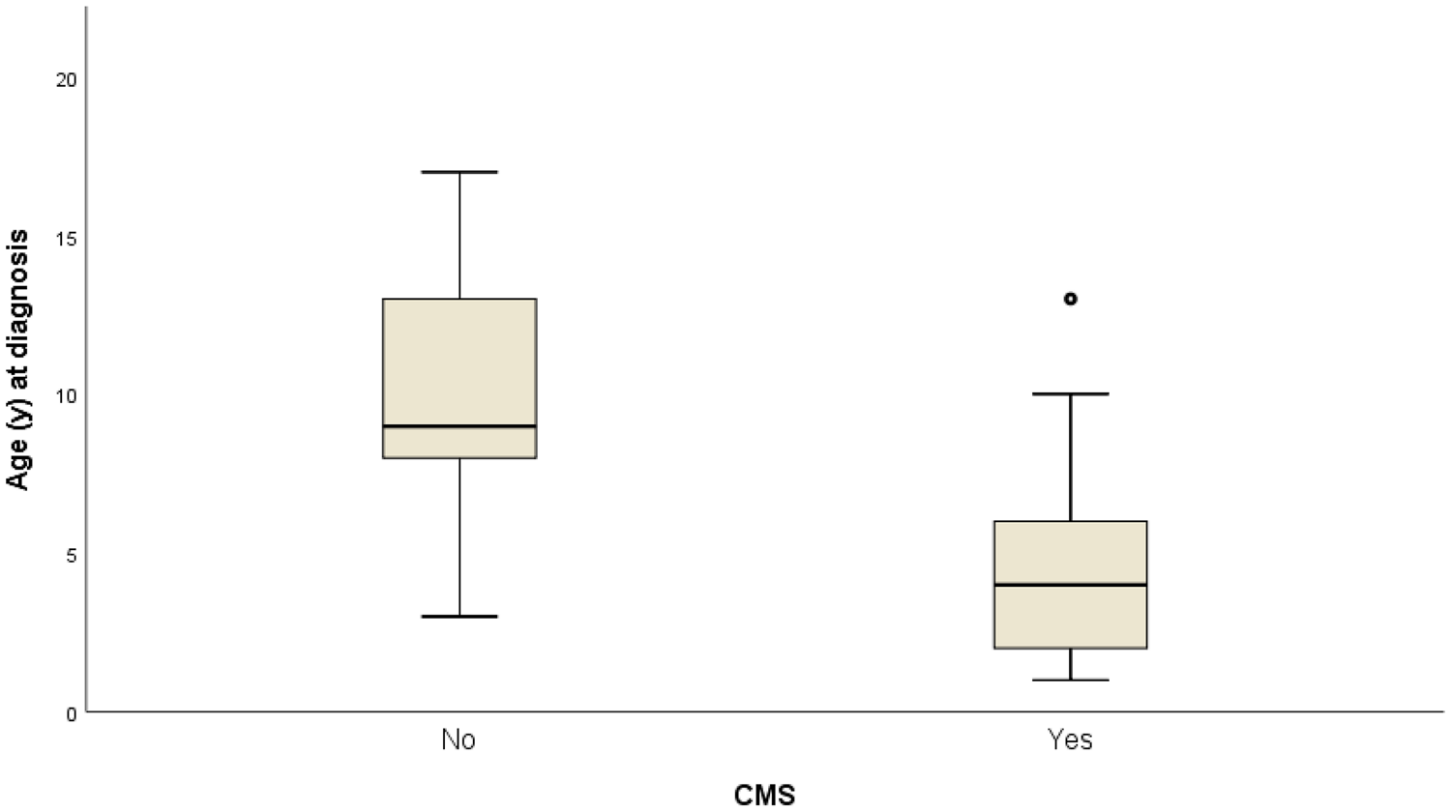
Side-by-side (comparative) box-plot of age at tumor diagnosis by cerebellar mutism syndrome (CMS). In the sample, patients without CMS (“No” CMS group) were older at time of tumor diagnosis compared with patients who developed CMS (“Yes” CMS group). The median age at tumor diagnosis for the Yes CMS group was 4 years old (q12-q38), compared to 9 years old (q17.5-q313) for No CMS group

Association analyses showed a significant difference in age at diagnosis: the occurrence of CMS was higher in younger patients (CMS group median 4 years of age, IQR 2–8) compared to the older ones (non-CMS group median 9 years, IQR 7–13) (*p* = 0.004, Mann–Whitney test: Fig. 2). Considering the dichotomization of the variable age at diagnosis (less than or equal to 5 years vs. over 5 years), there was a significantly higher frequency of CMS under 5 years of age (*p* = 0.008, Fisher’s exact test).

A higher frequency of CMS was found also in the subgroup of patients with a midline location of the tumor compared to other locations (*p* = 0.025, Fisher’s exact test). No statistically significant association was found with the other investigated variables (Table 1).

### Post-operative pediatric CMS scale

The spectrum of CMS symptoms in the study children varied from transient mutism without neurobehavioral symptoms to mutism with severe behavioral symptoms of longer duration. Mutism emerged immediately after surgery in 2 (15%) patients, after 1–2 days in 8 patients (62%) and after 2–4 days in 3 (23%) patients. The duration of the mutism varied from 5 days to 5 months. In 11 patients (85%), emotional lability was also present and lasted for more than 4 weeks in one patient, while in the others, it resolved within a month. Hypotonia (13 patients, 100%) was the most frequent and severe symptom, with the longest time to remission (more than a month). Dysphagia (9 patients, 69%) resolved within the first 25 days in 5 patients, although in 4 patients, it lasted for more than a month, with a gradual decrease in severity score.

Thirty days after the surgical resection (*T*_1_), the overall scales were scored. According to the CMS scoring system proposed, the intensity of the syndrome was scored mild in 4 (31%) patients, moderate in 4 (31%), and severe in 5 (38%). Detailed results are shown in Table 3.

**Table 3:** *T*_1_ CMS Survey scores

**Patient**	***T***_**0**_	**Mutism duration**	**Mutism severity**	**EL duration**	**EL severity**	**Hypotonia** **duration**	**Hypotonia** **severity**	**Dysphagia** **duration**	**Dysphagia** **severity**	**Total Duration** **Scale**	**Total Severity** **Scale**	**Overall Severity Scale**
1	2	2	3	2	2	3	3	3	2	3	3	3
2	2	3	3	0	0	3	3	3	3	3	3	3
3	1	2	1	0	0	3	3	3	3	3	3	3
4	2	1	2	2	1	3	3	1	1	2	2	2
5	2	2	1	2	1	2	2	0	0	2	1	1
6	2	3	3	3	3	3	3	0	0	3	3	3
7	2	1	1	2	1	2	2	2	1	2	1	1
8	2	2	2	1	1	2	3	1	1	2	2	2
9	1	1	1	2	1	3	3	2	1	2	1	1
10	2	1	2	1	1	2	2	0	0	1	2	1
11	3	3	3	3	1	3	3	3	2	3	3	3
12	3	2	2	2	1	3	3	0	0	2	2	2
13	3	2	2	2	3	3	3	2	3	2	3	2

*T*_0_ time of CMS onset, *T*_1_ assessment at day 30th after CMS onset, *EL* emotional lability (see Supplementary Material for the complete survey and scoring criteria)

The symptom scores on the “duration” and “severity” scales were rather homogeneous for each patient (the same or only 1-point discordant). When discordant, the duration score was higher than the severity score. A significant number of patients still had deficits after 1 month: 69% (9/13) had severe hypotonia, 23% (3/13) severe mutism, 31% (4/13) dysphagia, and 15% (2/13) emotional lability.

**Fig. 3:**
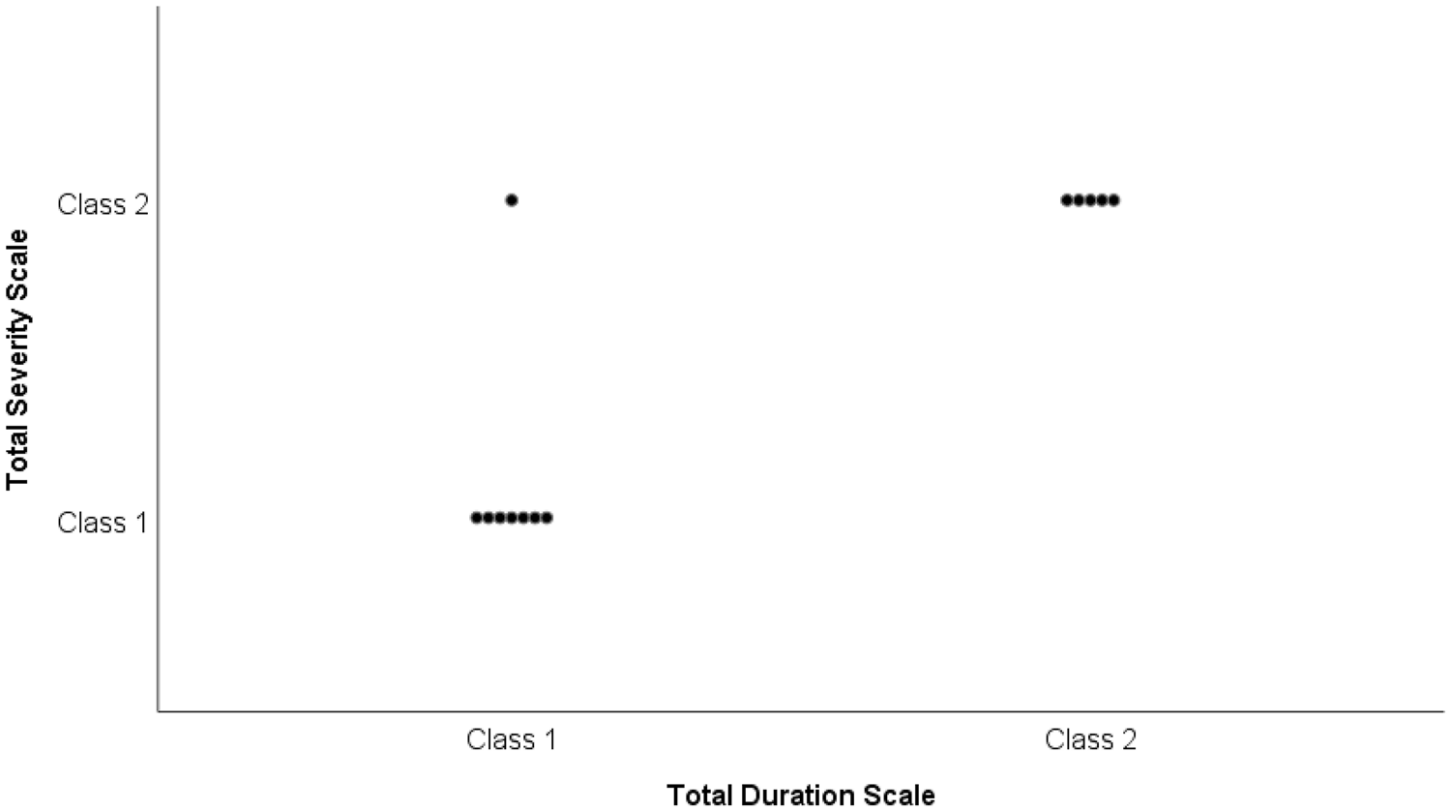
Duration and severity scatter-plot. “Total Severity Scale” and “Total Duration Scale” scores were each categorized into two severity classes: class 1 patients with moderate and mild scores (1/2), class 2 patients with severe scores. In the sample, higher scores on the Total Severity Scale corresponded to higher scores on the Total Duration Scale

Association analyses were performed to evaluate the possible clinical application of a symptoms’ severity scale, administered daily since the first day after surgery, to estimate syndrome duration. It emerged that higher scores on the “Total Severity Scale” corresponded to higher scores on the “Total Duration Scale” (*p* = 0.012, Fisher-Freeman-Halton test). After a further dichotomization of severity and duration into two classes (i.e., class one: patients with mild or moderate scores and class two: patients with severe scores), it was found that class 2 patients obtained higher scores on the Total Duration Scale compared to class 1 patients (*p* = 0.005, Fisher’s exact test: Fig. 3). Furthermore, a significant association between the Total Severity Scale scores at *T*_0_ and at *T*_1_ emerged, with higher severity scores at *T*_0_ corresponding to higher severity scores at *T*_1_ (*p* = 0.0001, Fisher-Freeman-Halton test).

## Discussion

The main goal of this work was to propose a diagnostic and scoring scale that could be used to identify and grade CMS. Following the recommendations emerged at the Consensus Meeting of the PFS in 2018, the proposed scale is brief, easily administrable, and based on a shared definition of CMS, including symptoms of mutism, emotional lability, hypotonia, and dysphagia. Formulating a scoring not only of the duration but also of the severity of these cardinal symptoms makes the scale very sensitive, allowing the diagnosis of even mild CMS presentations. The periodical compilation of the survey, starting from the immediate post-operative period, allows a prognostic stratification of patients to be made, which is fundamental for the consequent planning of appropriate rehabilitative and therapeutic interventions.

The data from the study sample showed that the scores of the Total Severity Scale and the Total Duration Scale were associated, with higher severity corresponding to longer duration. Even more interesting is the result that higher scores of Total Severity at *T*_0_ were associated with higher scores of Total Severity Scale at *T*_1_. If these results are confirmed by future studies with larger sample sizes, having a tool to estimate the severity of CMS since its initial presentation would allow an early prognostic stratification of patients. In particular, the early identification not only of patients with severe CMS, but also of those with mild or moderate CMS, would allow early intensive rehabilitative interventions to be promptly initiated to improve the overall prognosis [15].

We encountered greater difficulties with hypotonia, for which there is no standardized rating scale. Using a severity subdivision (e.g., mild/moderate/severe) would have made the score extremely subjective and difficult to compare between different observers. Therefore, we decided to grade it by defining the topographic extent of the hypotonia. This aspect could be improved in future refinements.

Although the scale seemed to be feasible for use in routine clinical practice, we have to note that a multiprofessional discussion was frequently needed to define the severity of the symptoms, especially hypotonia and dysphagia. A multiprofessional approach is also essential to optimize other factors that could influence emotional lability and language regression, especially in very young children (e.g., psychological support, pain relief). However, it may still be difficult to differentiate very mild cases of CMS from the effect of medications or discomfort. This point deserves further consideration and discussion between experts to arrive at a standardization.

A secondary aim of the work was to define the individual characteristics of patients suffering from CMS for prognostic categorization and stratification. In line with the risk factors already reported in the literature, patients who developed CMS were more likely to have a medulloblastoma, infiltration or compression of the brainstem, total tumor resection, or pre-operative hydrocephalus. However, these differences were not statistically significant, presumably due to the small sample of enlisted patients. The only variables found to have a statistically significant association with CMS were tumor location and age at diagnosis.

Another secondary aim was to investigate the diagnostic implications of the broader criteria for CMS, as suggested by the consensus meeting of the PFS [15]. Based on our sample, the estimated incidence was larger than most previously reported rates (43%, C.I. 95%: 25.5–62.6% in our sample vs. 11–30% in the literature) [6]. This difference could be due to the application of the scale in the immediate post-operative to evaluate not only patients with total absence of language but also those with milder symptoms that were considered sufficient for the diagnosis [13]. As proof of this, the prevalence of severe CMS in our sample was 17%, whereas the prevalence of CMS with moderate or severe symptoms was 30%, which is consistent with the data reported in the literature. It is therefore possible that the prospective compilation of the survey from the immediate post-operative involves greater diagnostic sensitivity, also including those patients with short-lived and mild intensity symptomatology that may not be detected retrospectively. As already reported, latency before onset of symptoms could be affected by sedation and mechanical ventilation after surgery.

Finally, because of the limited sample size, single site design, and the non-standardized scoring of the severity of symptoms, this study should be considered as a preliminary qualitative investigation aimed at exploring the utility and feasibility of the instrument. Indeed, future studies will be needed to quantitatively explore the sensitivity of this battery in detecting CMS and to validate the tool in a larger multicenter cohort of patients with PFT.

## Conclusions

The scale for diagnosis and scoring of post-operative CMS that was developed for this study, based on the latest definition from the 2015 Consensus Meeting of the PFS, proved to be simple, feasible, and applicable to clinical practice. The compilation of the data collection survey, carried out periodically starting from the immediate post-operative, makes the scale very sensitive, allowing the diagnosis of even mild CMS presentations. Formulating a scoring not only of the duration but also of the severity of cardinal symptoms can provide clinicians and researchers with a useful tool for prognostic stratification, which is critically important for optimal planning of the clinical-rehabilitative therapeutic activities. Thanks to the improvement of surgical techniques and co-adjuvant therapies available to treat children suffering from PFT, their survival rate is increasing, thus making it essential to plan for targeted rehabilitation interventions. The standardization of the diagnostic and prognostic criteria of CMS, and the development of a common clinical language between clinical centers and health professionals, can benefit both the clinical care of these patients and future research activities.

## Supplementary Information

Below is the link to the electronic supplementary material.

Supplementary file1 (DOCX 26 KB)

## Data Availability

Not applicable.

## Abbreviations

CMS: Cerebellar mutism syndrome
MRI: Magnetic resonance imaging
PFS: Posterior Fossa Society
PFT: Posterior fossa tumor
WHO: World Health Organization
