# Supplementary material for: Development and application of a diagnostic and severity scale to grade post-operative pediatric cerebellar mutism syndrome
Source: Eur J Pediatr. 2021 Oct 14;181(3):941–50. doi: 10.1007/s00431-021-04290-x (PMC8897365; doi:10.1007/s00431-021-04290-x)
Supplement: Supplementary file 1 — Supplementary file1 (DOCX 26 KB) [file 431_2021_4290_MOESM1_ESM.docx]

**OR1 Pediatric Post-Operative CEREBELLAR MUTISM SYNDROME (pCMS) Survey**

Unmet needs in clinical practice: a proposal and application of a diagnostic and severity scale to grade Cerebellar Mutism Syndrome, European Journal of Pediatrics, Federica S. Ricci¹; Rossella D’Alessandro¹; Alessandra Somà¹; Anna Salvalaggio¹; Francesca Rossi¹; Sara Rampone²; Giorgia Gamberini¹, Chiara Davico¹; Paola Peretta³; Mario Cacciacarne³; Pierpaolo Gaglini³; Paolo Pacca³; Giulia Pilloni³; Paola Ragazzi³; Daniele Bertin⁴; Stefano G. Vallero⁴; Franca Fagioli⁴; Benedetto Vitiello. federica.ricci@unito.it, Section of Child and Adolescent Neuropsychiatry, Children’s Hospital “Regina Margherita”

Name, Surname:

1. **MUTISM**

| Duration  1 = <1 week  2 = 1-4 weeks  3 = ≥ 4 weeks | Severity  1 = Short sentences  2 = Single words  3 = Total absence of verbal production  *For children <3 years consider quantitative reduction of the pre-operatory acquired language milestone or regression to a prior language milestone |
| --- | --- |

|  | DURATION (1, 2 or 3) | SEVERITY (1, 2 or 3) |
| --- | --- | --- |
| Day 1 |  |  |
| Day 2 |  |  |
| Day 3 |  |  |
| Day 4 |  |  |
| Day 5 |  |  |
| Day 6 |  |  |
| Day 7 |  |  |
| Day 8 |  |  |
| Day 9 |  |  |
| Day 10 |  |  |
| Day 11 |  |  |
| Day 12 |  |  |
| Day 13 |  |  |
| Day 14 |  |  |
| Day 15 |  |  |
| Day 16 |  |  |
| Day 17 |  |  |
| Day 18 |  |  |
| Day 19 |  |  |
| Day 20 |  |  |
| Day 21 |  |  |
| Day 22 |  |  |
| Day 23 |  |  |
| Day 24 |  |  |
| Day 25 |  |  |
| Day 26 |  |  |
| Day 27 |  |  |
| Day 28 |  |  |
| Day 29 |  |  |
| Day 30 |  |  |

1. **EMOTIONAL LABILITY**

| Duration  1 = <1 week  2 = 1-4 weeks  3 = ≥ 4 weeks | Severity  1 = Emotionality congruous / low threshold, containable states of agitation  2 = Emotional sometimes congruous and sometimes incongruous / low-threshold, poorly contained agitation states  3 = Incongruous emotionality / incongruous states of agitation |
| --- | --- |

|  | DURATION (1, 2 or 3) | SEVERITY (1, 2 or 3) |
| --- | --- | --- |
| Day 1 |  |  |
| Day 2 |  |  |
| Day 3 |  |  |
| Day 4 |  |  |
| Day 5 |  |  |
| Day 6 |  |  |
| Day 7 |  |  |
| Day 8 |  |  |
| Day 9 |  |  |
| Day 10 |  |  |
| Day 11 |  |  |
| Day 12 |  |  |
| Day 13 |  |  |
| Day 14 |  |  |
| Day 15 |  |  |
| Day 16 |  |  |
| Day 17 |  |  |
| Day 18 |  |  |
| Day 19 |  |  |
| Day 20 |  |  |
| Day 21 |  |  |
| Day 22 |  |  |
| Day 23 |  |  |
| Day 24 |  |  |
| Day 25 |  |  |
| Day 26 |  |  |
| Day 27 |  |  |
| Day 28 |  |  |
| Day 29 |  |  |
| Day 30 |  |  |

1. **HYPOTONIA**

| Duration  1 = <1 week  2 = 1-4 weeks  3 = ≥ 4 weeks | Severity  1 = Ipsilateral  2 = Affects the four limbs  3 = Affects the four limbs and the axis |
| --- | --- |

|  | DURATION (1, 2 or 3) | SEVERITY (1, 2 or 3) |
| --- | --- | --- |
| Day 1 |  |  |
| Day 2 |  |  |
| Day 3 |  |  |
| Day 4 |  |  |
| Day 5 |  |  |
| Day 6 |  |  |
| Day 7 |  |  |
| Day 8 |  |  |
| Day 9 |  |  |
| Day 10 |  |  |
| Day 11 |  |  |
| Day 12 |  |  |
| Day 13 |  |  |
| Day 14 |  |  |
| Day 15 |  |  |
| Day 16 |  |  |
| Day 17 |  |  |
| Day 18 |  |  |
| Day 19 |  |  |
| Day 20 |  |  |
| Day 21 |  |  |
| Day 22 |  |  |
| Day 23 |  |  |
| Day 24 |  |  |
| Day 25 |  |  |
| Day 26 |  |  |
| Day 27 |  |  |
| Day 28 |  |  |
| Day 29 |  |  |
| Day 30 |  |  |

1. **OROPHARYNGEAL DYSPHAGIA**

| Duration  1 = <1 week  2 = 1-4 weeks  3 = ≥ 4 weeks | Severity  1 = Liquid dysphagia  2 = Dysphagia for chewing foods  3 = Complete dysphagia |
| --- | --- |

|  | DURATION (1, 2 or 3) | SEVERITY (1, 2 or 3) |
| --- | --- | --- |
| Day 1 |  |  |
| Day 2 |  |  |
| Day 3 |  |  |
| Day 4 |  |  |
| Day 5 |  |  |
| Day 6 |  |  |
| Day 7 |  |  |
| Day 8 |  |  |
| Day 9 |  |  |
| Day 10 |  |  |
| Day 11 |  |  |
| Day 12 |  |  |
| Day 13 |  |  |
| Day 14 |  |  |
| Day 15 |  |  |
| Day 16 |  |  |
| Day 17 |  |  |
| Day 18 |  |  |
| Day 19 |  |  |
| Day 20 |  |  |
| Day 21 |  |  |
| Day 22 |  |  |
| Day 23 |  |  |
| Day 24 |  |  |
| Day 25 |  |  |
| Day 26 |  |  |
| Day 27 |  |  |
| Day 28 |  |  |
| Day 29 |  |  |
| Day 30 |  |  |
